# Facilitating multidisciplinary working groups in translational research: Strategies to promote cross-center collaboration and sustain the Cancer Center Cessation Initiative Consortium

**DOI:** 10.1017/cts.2024.653

**Published:** 2024-11-15

**Authors:** Mara Minion, Betsy Rolland

**Affiliations:** 1 Center for Tobacco Research & Intervention, University of Wisconsin-Madison, Madison, WI, USA; 2 Carbone Cancer Center, University of Wisconsin-Madison, Madison, WI, USA; 3 Institute for Clinical and Translational Research, University of Wisconsin-Madison, Madison, WI, USA; 4 Michigan Institute for Clinical & Health Research, University of Michigan, Ann Arbor, MI, USA

**Keywords:** Working groups, team science, collaboration, best practices

## Abstract

As funding for large translational research consortia increases across the National Institutes of Health (NIH), focused working groups provide an opportunity to leverage the power of unique networks to conduct high-impact science and offer a strategy for building collaborative infrastructure to sustain networks long-term. This sustainment leverages the existing NIH investments, amplifying the impact and creating conditions for future innovative translational research. However, few resources exist that detail practical strategies for establishing and sustaining working groups in consortia. Here, we describe how the Coordinating Center for the National Cancer Institute-funded Cancer Center Cessation Initiative (C3I) utilized principles derived from the Science of Team Science to develop replicable strategies for building and sustaining an effective working group-led consortium. These strategies include continually engaging community members in strategic planning, prioritizing diversity in leadership and membership, creating multi-level opportunities for leadership and participation, providing intensive community management and facilitation, and incentivizing projects that support the consortium sustainment. When assessing the impact of these interventions through qualitative exit interviews, four key themes emerged: through the C3I working group consortium, members co-created new dissemination products, gained new insights and innovations, enhanced local program implementation, and invested in cross-network collaboration to support sustained engagement in the initiative.

## Background

As funding for large translational research consortia increases across the National Institutes of Health (NIH), focused working groups provide an opportunity to leverage the power of these unique networks to conduct high-impact science and offer a strategy for building collaborative infrastructure to sustain successful networks long-term. Several high-profile translational research consortia exist, including the National Clinical Cohort Collaborative (NC3), the Population-based Research to Optimize the Screening Process (PROSPR), and Brain Research Through Advancing Innovative Neurotechnologies® Initiative (BRAIN Initiative®), all of which have working groups aimed at advancing specific aspects of the initiative’s research agenda. However, few resources exist that detail best practices and practical strategies for establishing and sustaining working groups in scientific consortia. Working groups can be forums for team science, as researchers work together, frequently across disciplines, to generate new knowledge. As such, we can apply principles of effective collaboration, derived from the Science of Team Science (SciTS) literature [1], adapted through our lens of experience building and leading Coordinating Centers [2–7], to the facilitation of multidisciplinary working groups in translational research. Here, we describe the impact of applying such principles to promote collaboration and sustain the Cancer Center Cessation Initiative (C3I) Consortium.

### The Cancer Center Cessation Initiative (C3I)

**Figure 1.**
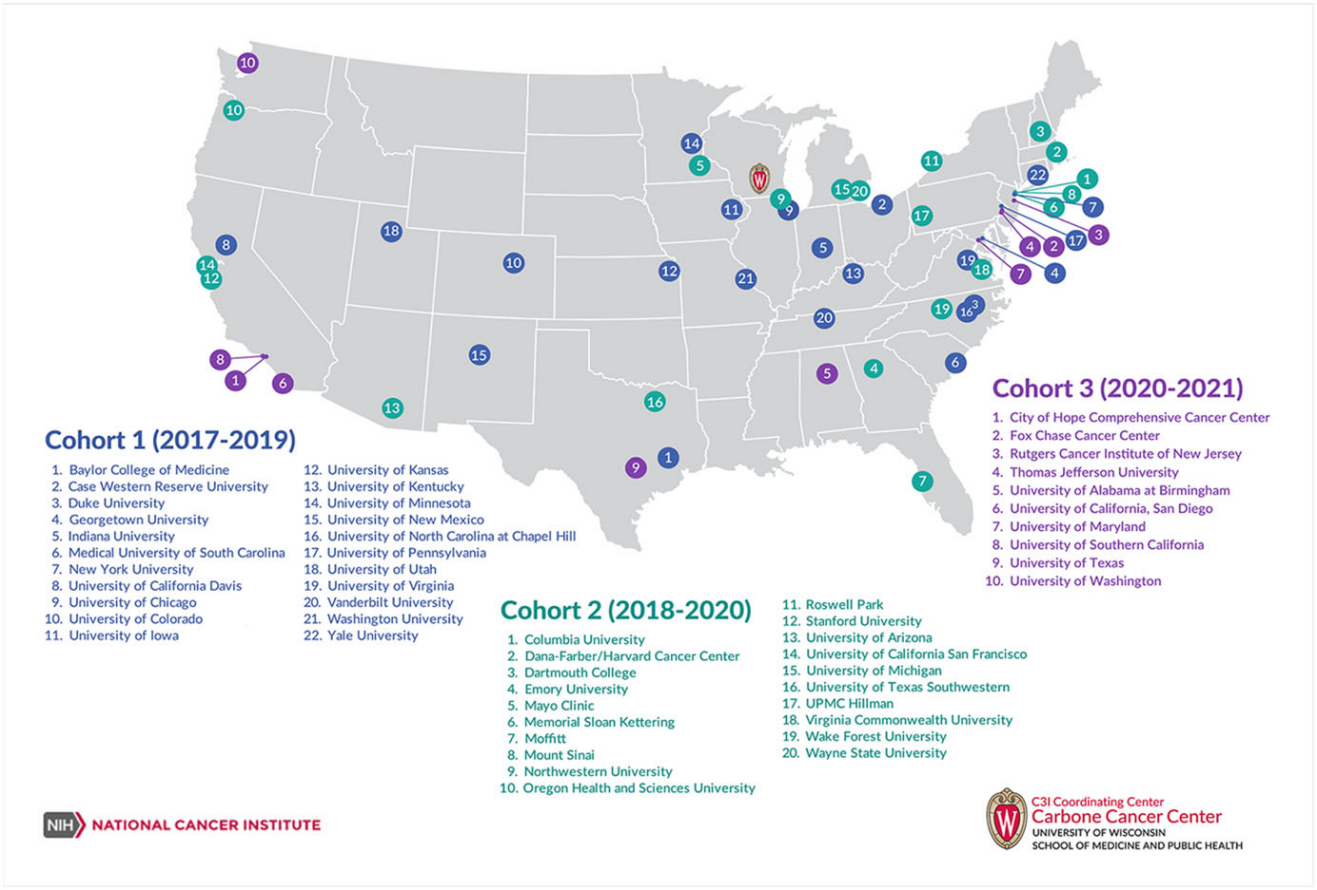
Cancer centers participating in Cancer Center Cessation Initiative as part of the National Cancer Institute cancer Moonshot^SM^ program.

In 2017, the National Cancer Institute (NCI) launched the C3I as part of the Cancer Moonshot^SM^ program to address the lack of routine tobacco use assessment and tobacco cessation treatment in oncology settings for cancer patients who smoke. The goals of the initiative were to implement and integrate evidence-based tobacco treatment services into clinical care at funded cancer centers and disseminate lessons learned to the cancer care community, establishing tobacco cessation as a fourth pillar of cancer care [1,2]. Over five years, three successive cohorts of grantees were funded, eventually including 52 of the 64 eligible clinical and comprehensive NCI-designated cancer centers (Figure 1). Funded as administrative supplements to each site’s Cancer Center Support Grant, C3I required centers to take a population-based approach to ensure that every patient with cancer is screened for tobacco use and that every individual who smokes receives evidence-based cessation treatment.

The C3I Coordinating Center, housed at the University of Wisconsin Carbone Cancer Center, supported C3I centers by providing technical assistance in the areas of electronic health record (EHR) integration and clinical workflow change management; collecting data on reach and effectiveness to monitor the impact of the initiative; serving as the hub of knowledge integration and dissemination; and developing and sustaining a collaborative consortium of tobacco researchers, tobacco treatment specialists, program managers, and clinicians. Co-led by experts in team science and tobacco cessation, the Coordinating Center aimed to develop a community of researchers engaged in collaborative learning and sharing. This community-led collaboration facilitated by the centralized Coordinating Center enabled funded sites to create and utilize a network of learning to surpass what each could achieve individually and increase the collective impact of this multi-site research initiative.

While working groups are common in large research consortia such as C3I, few resources exist to guide their formation, facilitation, management, or evaluation. The terms “committee,” “special interest group,” and “working group” are often (incorrectly) used interchangeably, without regard for the group’s overarching goals. Those goals, success metrics, and governance structures often go unspecified, leaving participants confused about why the group exists and what is required of participants. This dearth of resources leads each consortium to reinvent the wheel, basing facilitation decisions on previous experience rather than on evidence. For the C3I Working Groups, we use the definition of Working Groups from the Center for Scientific Collaboration and Community Engagement (CSCCE) that posits key characteristics, including a defined scope, clear intended outputs, and regular meetings to advance the group’s goals [8]. (For further information, please also see [9].) Here, we present five replicable strategies based on principles from the Science of Team Science that consortia can implement to increase engagement with working groups.

## C3I principles for facilitating collaboration

From the beginning of the initiative, the Coordinating Center adopted an evidence-based, team-science approach to facilitated collaboration, with a primary focus on four key principles derived from the SciTS research [1,4–6,10]:

1. Facilitate a culture of openness and collaborative learning
2. Invest in systematic, continuous learning across C3I cohorts
3. Collect data to monitor and report progress to participating centers
4. Identify and provide resources to meet centers’ needs

**Figure 2.**
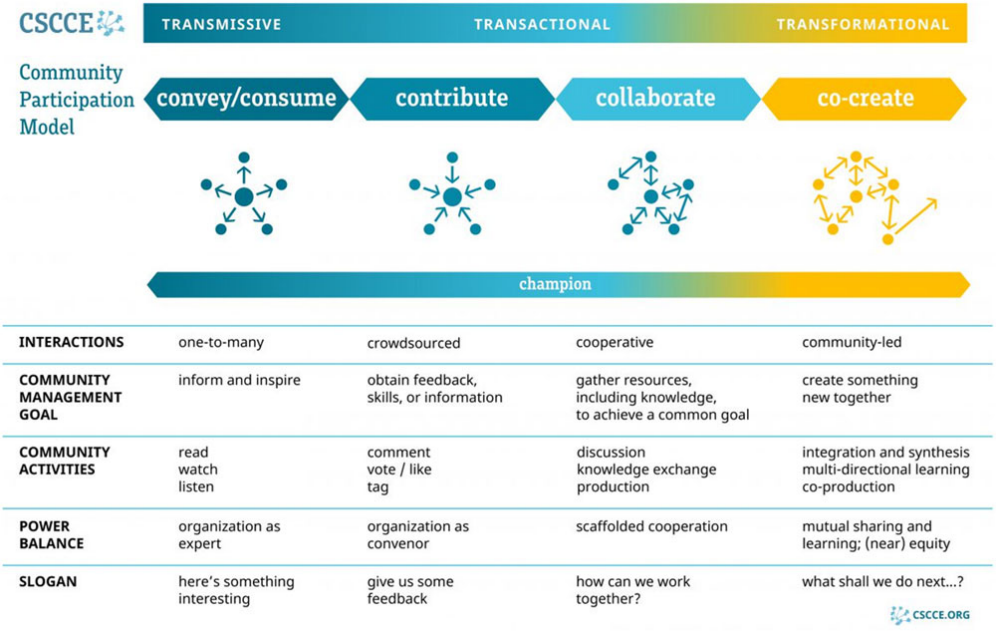
The Center for Scientific Collaboration and Community Engagement community participation model [3].

**Figure 3.**
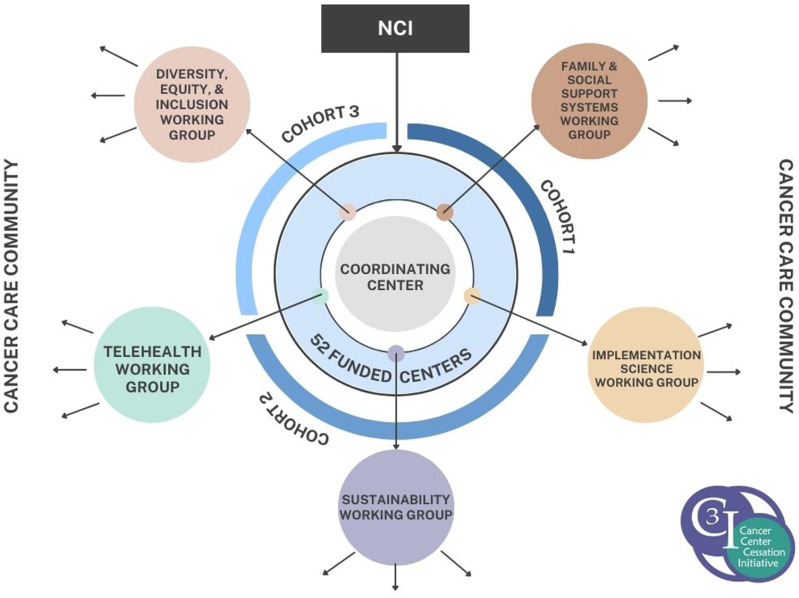
Cancer Center Cessation Initiative working group consortium sketch.

These principles have guided Coordinating Center programing, proved successful for community building, and informed strategies to shift the initiative into a self-sustaining consortium to continue to generate common data collection and lessons learned to advance the science of tobacco cessation in cancer care. Using the CSCCE Community Participation Model [11], we can map this programing onto the model to understand the modes of C3I community members’ participation and ensure we are fostering diverse and impactful interactions, activities, and outcomes (Figure 2). Through regular all-grantee meetings, webinars, EHR consultations, a mentorship program, shared portal of resources, and discussion forum, C3I grantees have been able to receive guidance and best practices, contribute their own strategies and lessons learned, and collaboratively exchange resources and knowledge to successfully achieve the first goal of the initiative: translating evidence-based tobacco treatment services into clinical care. To more effectively address the second goal of the initiative – disseminate lessons learned to the cancer care community to establish tobacco cessation as a pillar of cancer care – and to support participation in the “co-create” mode of the CSCCE Community Participation Model (Figure 2), in 2020, the Coordinating Center established a grantee-led working group consortium (Figure 3).

## Strategies for building a consortium

**Figure 4.**
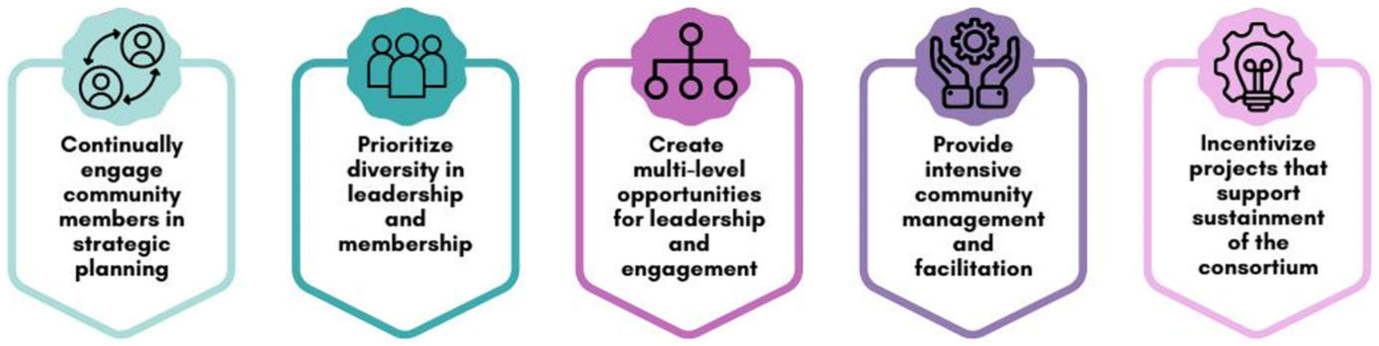
Cancer Center Cessation Initiative coordinating center strategies for facilitating working groups.

Our aim was to build infrastructure through which C3I community members could capture collective knowledge to advance the science of tobacco cessation by collaboratively generating new cross-site projects, publications, funding proposals, and additional resources that leverage the existing C3I network. To accomplish this aim, we implemented five replicable strategies to build and sustain a robust, engaged, and productive scientific community (Figure 4): (1) continually engage community members in strategic planning, (2) prioritize diversity in leadership and membership, (3) create multi-level opportunities for leadership and participation, (4) provide intensive community management and facilitation, and (5) incentivize projects that support the sustainment of the consortium. Here, we recount our implementation of these strategies through a number of activities. While we believe the strategies are replicable for any scientific community, the activities should be designed with the specific community needs and context in mind. As such, the activities described here can serve as examples, rather than prescriptions.

### Continually engage community members in strategic planning

In Spring 2020, the Coordinating Center surveyed C3I grantees, soliciting their priority research questions that could be uniquely addressed by a sustained C3I consortium. We categorized the responses into five key themes and formed the C3I working groups around those grantee-identified areas of focus: (1) diversity, equity, and inclusion; (2) family and social support systems; (3) implementation science; (4) sustainability; and (5) telehealth. Building the groups around the shared priorities of the C3I community members ensured their strong investment in the consortium from the beginning.

The Coordinating Center continues to seek community input in ongoing high-level decision-making for the consortium. We meet annually with the working group leadership and regularly survey membership in order to reflect on the successes and challenges of the groups, identify future goals, and re-align strategic planning to support those goals. For example, in an annual leadership meeting, the Coordinating Center requested suggestions for ways to better facilitate cross-group collaboration. Chairs suggested establishing a regular webinar series in which each individual group could share their work with the broader community and engage collaborators from other groups. The Coordinating Center then surveyed all members on preferences for the content, frequency, and scheduling of this series and implemented bimonthly working group webinars as consortium programing. Soliciting regular input from working group leadership and membership is key to developing relevant resources to reduce barriers and challenges and adapting the infrastructure of the consortium to meet the evolving needs of the community members.

### Prioritize diversity in leadership and membership

The Coordinating Center identified two or three co-chairs per group and invited those C3I members to serve, prioritizing demographic and disciplinary diversity and pairing early-career investigators with established investigators when possible. In addition, we ensured that all three C3I cohorts were represented in the leadership of the groups. Research has shown that heterogeneity in science teams can increase their productivity, impact, and capacity to address complex problems [12]. As such, our goal was to support as much diversity as possible in both the leadership and membership, across multiple facets.

**Figure 5.**
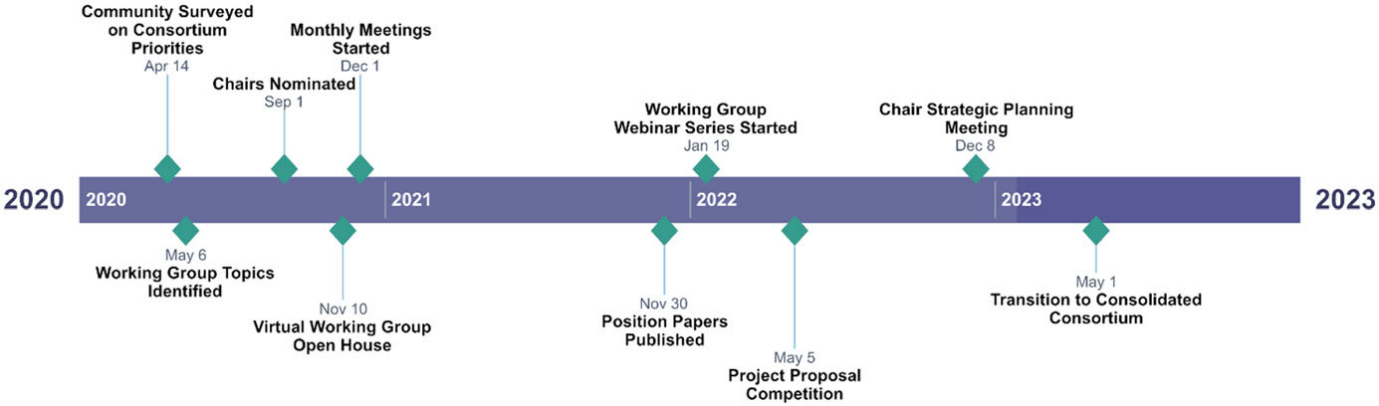
Cancer Center Cessation Initiative working group consortium timeline.

The Coordinating Center met with the co-chairs of each group to identify preliminary goals for projects that addressed tobacco cessation research, practice, and/or policy. Our aim in doing so was to meet the professional interests and needs of C3I community members across disciplines, including researchers, clinicians, tobacco treatment specialists, and project managers. In Fall 2020 at the C3I all-grantee meeting, each working group’s co-chairs presented their priorities and plans in a virtual “open house” format to recruit working group members, and monthly meetings began in December 2020 (Figure 5).

Since that first recruitment, engagement with the working groups has been robust. To date, 110 C3I members have participated, with 38 individuals engaged with multiple groups. Of the 52 funded centers, 45 are represented in the groups. This multidisciplinary consortium consists of 42 C3I program leads, 23 tobacco treatment specialists, 16 clinical collaborators, 13 project managers, 7 students and research assistants, 2 IT staff, and 7 non-C3I collaborators (e.g., researchers from non-funded centers). The working group membership reflects the disciplinary diversity of the C3I initiative, and the diverse perspectives and investments of the members sustain the consortium’s focus to broadly impact the entire cancer care community through research, practice, and policy.

### Create multi-level opportunities for leadership and participation

The Coordinating Center established shared and staggered leadership of the working groups by nominating multiple co-chairs per group. Each chair commits to serving at least one year with the option to serve for multiple years. Once a chair opts to rotate out, any working group member can apply for the open leadership position by completing a brief survey detailing their ideas and goals for the group. The Coordinating Center and continuing chairs then select a new chair from the applicants together. This model promotes sustainability: the co-chair system shares the responsibilities of leadership across multiple individuals, and the staggered rotation maintains some continuity of leadership year-to-year while also creating space for new leaders to take on a chair role. While our co-chairs did not experience any disagreements while leading WGs, our WG charter did lay out a scope for how to address disagreements internally and request mediation from the Coordinating Center when necessary.

In addition, we have created multiple opportunities for leadership and engagement outside of the chair positions. While the chairs facilitate the groups and guide the agenda for meetings, any working group member can lead an individual project or publication. Similarly, while the chairs organized and hosted the webinars in our 2022 working group webinar series, other working group members presented on group activities or relevant interventions at their own centers. These multi-level leadership opportunities are key to incentivizing robust engagement from the full membership, distributing burden and effort across many individuals, and preventing working group activities from becoming dependent on a small number of highly active members.

### Provide intensive community management and facilitation

The Coordinating Center dedicated personnel to provide intensive community management for the C3I working groups. The Coordinating Center Project Manager (MM) serves as a community manager for the consortium, onboarding new members, scheduling and facilitating all meetings, tracking detailed minutes and action items, maintaining community resources and platforms, and managing communications. By developing scaffolding resources, tracking progress toward and providing administrative support for deliverables, and facilitating cross-group collaborations, the Project Manager functions as a central node, building infrastructure and weaving connective threads to strengthen the network.

The CSCCE defines scaffolding as, “the supportive information, activities, and processes that address barriers to member participation and ensure all members can access and engage in community programming.” [11] In addition to reducing barriers to entry, scaffolding promotes more effective team functions and processes by setting bounded, interdependent roles while creating a sense of collective responsibility for group members [13]. The Coordinating Center Project Manager created scaffolding to support community member participation and integration in the C3I working groups, including a website and sign-up form for the groups, onboarding surveys for new members, community participation guidelines through a Roles & Responsibilities charter, group contact lists, technical guides for the document-sharing platform, a table of contents for the resource library, as well as templates for meeting materials such as agendas, minutes, and slides. These scaffolds remove barriers to participation in the groups, alleviate the burden of leadership for the co-chairs, and provide infrastructure to promote investment in the collective and sustain working group activities when administrative support from the Coordinating Center ends (currently projected to be Fall 2025).

Our previous experience has shown that working groups are most successful when they have a tangible focus for their energy and efforts. As such, the Coordinating Center identified a strategic “low-hanging fruit” for the first co-created C3I working group deliverable: a collection of position papers with an introduction from the Coordinating Center, published as a supplement in the Journal of the National Comprehensive Cancer Network in November 2021 [14]. Each working group contributed a short paper that explained the importance of each group’s focus on tobacco cessation in cancer patients who smoke, characterized the relevant work being done in C3I and outlined a research agenda for each group’s topic. These agendas inform a tracking document used to monitor progress toward the groups’ proposed deliverables. The Coordinating Center created collective authorship guidelines, drafting schedules, and review processes, and the Project Manager provided significant administrative support to facilitate manuscript development and submission across the five groups. This first project was a “quick win” that catalyzed collaboration and generated momentum for continued activity and direction for future projects.

Through the five working group topics – diversity, equity, and inclusion; family and social support systems; implementation science; sustainability; and telehealth – there are many cross-cutting themes and shared priorities. The Coordinating Center aims to promote synergy rather than redundancy with the Project Manager serving as a connecting thread for the groups. Facilitating each meeting enables the Project Manager to regularly seek out and identify opportunities for cross-working group collaboration, connect members in different groups with shared interests and project ideas, and invite groups to consolidate similar efforts, such as data collection. The Project Manager facilitates community connections and productive collaborations to effectively leverage the potential of the full network.

### Incentivize projects that support the sustainment of the consortium

To further catalyze the development of new cross-network projects, the Coordinating Center launched a cross-working group project proposal competition in Spring 2022. All working group members were invited to submit collaborative proposals for projects that engaged collaborators from multiple groups, with special consideration for projects with a health equity focus. Eligible submissions were selected for presentation at the virtual C3I cross-working group meeting in May 2022. For this “Shark Tank”-like event, each project lead pitched their proposal before a panel of judges, including NCI program directors and other tobacco cessation experts, and received feedback for the revision and final submission of the proposal for consideration in the competition. Five exemplary project proposals were selected for small pilot awards from the Coordinating Center to support the development of pilot studies and initial products over the next year with the ultimate goal to produce new federal funding proposals that will help sustain the consortium.

## Outcomes

In June 2022, the Coordinating Center conducted 30-min virtual exit interviews with the leadership team of each of the 10 Cohort 3 centers. The purpose of the interviews was to obtain in-depth perspectives on the impact of Coordinating Center programing and the C3I network as whole on program implementation. The interviews were categorized as program evaluations and deemed exempt by the University of Wisconsin-Madison Institutional Review Board. The interview guide (Supplementary Material) included four main questions with additional probes to better understand the effects of Coordinating Center resources, interactions with other community members, and specific implementation strategies on the program and cancer center. When describing what types of interactions with other grantees helped to effectively design and implement their programs, six (60%) of the centers mentioned the working groups as high-impact programing, and four main themes regarding that impact emerged:

### Co-created dissemination products

Respondents referenced the many dissemination products co-created through the working groups, including publications and presentations, as key deliverables of their involvement that added value within their own programs as well. This program manager highlighted that these dissemination products continue to be circulated as resources within their center:

*“We were able to get two papers completed and then, on top of that, a webinar presentation, highlighting, you know, DEI in our center. I thought all that was really helpful for our program as well, because then we have slide decks and materials we can share within house.”* (Respondent 2, Center 3).

Dissemination products are the currency of academic research; providing high-impact, low-burden opportunities for working group members to participate in such products keeps them invested and showing up.

### Gained new insights and innovations from informal discussions

In addition to publications and presentations, respondents noted that informal knowledge co-creation through discussions at working group meetings was also highly valuable. This clinical collaborator explained:

*“They were the informal development of concepts and ideas that you want to put in place at your institution. I think these are very important because it’s truly through these discussions that innovations come to mind, and these innovations help to take us forward in a much more rapid process than waiting to read someone else’s paper that was just published. Also, you find out the background behind the research that’s been done so far.”* (Respondent 3, Center 1).

The working groups created space for informal conversations among colleagues that ultimately led to new ideation in both implementation and research.

### Enhanced program implementation

Importantly, grantees were able to learn from the experiences and expertise of the groups as a whole in order to enhance their own program implementation, thus furthering NCI’s aims in establishing C3I. This tobacco treatment specialist noted the ways in which the Family and Social Support Systems Working Group changed their clinical practice:

*“[Engaging family members] was one piece that I believe that we were missing within our program itself, so when we’re talking with patients and engaging them, it’s important to also address the family needs and if there are additional smokers within the home and how we can help those individuals.”* (Respondent 2, Center 2).

This program leader volunteered that the working groups influenced their sustainability plans for their program:

*“You got to pick up things from other sites and apply them…the sustainability efforts, I mean, that was really helpful, and we have now two years of sustainability funding, and I think we learned a lot from hearing from others how they approached that to help in that process.”* (Respondent 1, Center 2).

In this way, the consortium not only led to the co-creation of new knowledge but also facilitated other modes of knowledge exchange and community participation to improve local implementation at individual sites.

### Connected to a community, especially beyond initial funding

Multiple respondents cited a sense of belonging and a connection to the community as a benefit of the working groups and an incentive to remain involved even after the end of the funded period. This program manager noted:

*“The work groups…they’re like smaller groups, and then within the work groups kind of had like groups of people working on different things, and so we really felt part of the team with the DEI work and the implementation science work.”* (Respondent 2, Center 3).

This program leader asserted their intention to remain engaged long-term:

*“I will continue to attend the meetings and be involved in any way that I can because I’m learning things there, in general, and I want to stay connected, you know, to the group even though, you know, after our funding has ended.”* (Respondent 1, Center 5).

This feedback touches on the importance of effective community management and fostering relationships for sustained member engagement. The C3I working groups were formed as the funding period of the initiative was ending, meaning all working group leadership, participation, and activities require entirely volunteer work from community members. Individuals tend not to collaborate on unpaid projects with people they do not know, like, or trust, and building relationships of trust in a professional network requires interpersonal skills, cultural and structural competency, and ongoing mediation [10]. This work is often relegated to the feminized and devalued category of “soft skills.” [15] However, these so-called “soft skills” are essential for creating a productive consortium, and affect-based trust in leadership can be positively linked to psychological safety and team performance [16]. While the small talk at the start of meetings or the inside joke circulated in the meeting minutes may seem relatively inconsequential, the work of relationship-building impacts the quality, quantity, and sustainability of a scientific network’s output [17,18].

## Future directions

From the beginning, the Coordinating Center aimed to build a C3I consortium that could be sustained long-term, beyond the planned funding from the NCI, and implemented the above strategies to support that sustainability. In late 2022, we solicited input from the leadership and membership on strategic planning and adaptations for sustaining the groups once Coordinating Center support ends as the initiative moves toward its planned closure.

Based on feedback, to address anticipated barriers of lower member bandwidth and decreased administrative support due to the winding down of the initiative by the NCI, the Coordinating Center consolidated the five working groups into a single collective that meets monthly to discuss all special interests and ongoing projects. Each project group meets separately with just the active collaborators for that particular work. Chairs continue to facilitate the meetings and maintain a focus on each of the five priority topics (diversity, equity, and inclusion; family and social support systems; implementation science; sustainability; telehealth). The Coordinating Center has developed new infrastructure, resources, platforms, meeting schedules, and member roles and responsibilities to facilitate the transition to this new organizational structure. This reorganization had several benefits, including reducing the number of monthly meetings, particularly for the members engaged in multiple groups; lessening the administrative burden of maintaining five separate groups and distributing community management responsibilities across multiple chairs and project leads; and creating dedicated venues for achieving project-specific deliverables and for sharing with a larger group to identify connections and potential collaborations.

When surveyed, the majority of respondents were in favor of the shift, with many noting that the reorganization would increase the likelihood of their participating in the consortium long-term. Through both survey responses and meetings with the chairs and members, the Coordinating Center received multiple comments emphasizing the value of the C3I consortium thus far and the importance of sustaining this collaborative community. As one respondent asserted, “If we lose this, we will never get this type of group together again. The value of this consortium is immense and honestly very early in its impact.” The urgency of this statement will guide future directions and adaptations to expand and extend the impact of the C3I working group consortium beyond the funded initiative.

## Conclusion

In large, multi-site translational research initiatives, working groups have the potential to facilitate high-impact team-based research and provide infrastructure to sustain successful networks. This sustainment leverages the existing investments made by NIH, amplifying the impact of those dollars and creating conditions for future innovative translational research. Informed by team-science principles, the C3I Coordinating Center developed five strategies for establishing an effective working group consortium and building sustainable systems to support the consortium long-term. These strategies included: (1) continually engaging and reengaging community members in strategic planning, (2) prioritizing diversity in leadership and membership, (3) creating multi-level opportunities for leadership and participation, (4) providing intensive community management and facilitation, and (5) incentivizing projects that support the sustainment of the consortium. These strategies helped cultivate the co-creation of new dissemination products and innovation, the enhancement of local implementation, and the collective sense of belonging and investment in the C3I community. This approach can be replicated and tailored in other scientific consortia to enhance and sustain community engagement, collaboration, and impact.

## Supporting information

Minion and Rolland supplementary material Minion and Rolland supplementary material

## Supplementary material

The supplementary material for this article can be found at https://doi.org/10.1017/cts.2024.653.
