# Supplementary material for: Facilitating multidisciplinary working groups in translational research: Strategies to promote cross-center collaboration and sustain the Cancer Center Cessation Initiative Consortium
Source: J Clin Transl Sci. 2024 Nov 15;8(1):e216. doi: 10.1017/cts.2024.653 (PMC11713431; doi:10.1017/cts.2024.653)
Supplement: Minion and Rolland supplementary material [file S2059866124006538sup001.docx]

**Supplementary Material**

**C3I Exit Interview Guide**

1. Which C3I resources were most valuable for effectively designing and implementing your program? *Examples of C3I resources: Patient materials, provider materials, EHR guides (billing/coding, eReferral, scripting, flowsheets, sample language, build guide), EHR consulting services, data reporting guides, background literature, training resources, portal forum, and webinars*
2. What types of interactions with other grantees helped you effectively design and implement your program? *Examples of interactions could include mentor/mentee sites, scientific meetings and breakout groups, working groups, portal and/or email discussions.*
   - - - 1. What is one thing you learned from interaction from other grantees that impacted how you operated your program?
3. What was the best organizational or scientific decision you made during this project to effectively design and implement your program? How do you think that decision will impact the longer-term sustainability of your program?
4. What impact did this initiative have on your Cancer Center?
